# Indian culture and psychiatry

**DOI:** 10.4103/0019-5545.69259

**Published:** 2010-01

**Authors:** Shiv Gautam, Nikhil Jain

**Affiliations:** Department of Psychiatry, Sr. Professor, Superintendent, Addl. Principal, SMS Medical College, Jaipur - 302 004, India; 1 2^nd^ yr. Resident Doctor, Psychiatric Centre, SMS Medical College, Jaipur - 302 004, India

**Keywords:** Culture & Psychiatry, culture bound syndromes, belief system

## Abstract

‘Culture’ is an abstraction, reflecting the total way of life of a society. Culture uniquely influences mental health of people living in a given society. Similarity in thinking and understanding of mental health across the ancient cultures has been observed. Studies which relate to the demographic factors, cultural factors influencing presentation of illness, diagnosis of the illness-culture bound syndromes and influence of the cultural factors and the belief system on psychopathology, stigma and discrimination towards the patient have been reviewed. An attempt has been made to critically look at the research on culture and psychiatry in different areas. There is a need for culturally oriented modules of non-pharmacological management.

## INTRODUCTION

‘Culture’ is an abstraction, reflecting the total way of life of a society. It is a precipitate of the group’s history and an expression of its adaptation to the physical environment. It refers to the shared patterns of beliefs, feeling and behavior and the basic values and concepts that members of the group carry in their minds as guides for the conduct. Besides social relationships, economics, religion, philosophy, mythology, scriptures, technology and other aspects of living contribute to the culture. Culture is constantly in the process of change and it is transmitted from one generation to the next. All societies have it though their styles vary from one group to another.[1]

The term “culture”, which is a keystone in psychiatry, is plagued by confusion because of a lack of concise, universally acceptable definition. In fact at least one hundred and sixty different definitions exist. Culture is thus best conceptualized as a totality, composed of a complex system of symbols possessing subjective dimensions such as values, feelings, and ideals and objective dimensions including beliefs, traditions, and behavioral prescriptions, articulated into laws and rituals. This unique capacity of culture to bind the objective world of perceived reality to the subjective world of the personal and intimate, lends it, its powerful role as expressor, mediator, and moderator of psychological processes and, ultimately, emotional disorders.[2]

Culture uniquely influences mental health of people living in a given society. Mental health problems, from presentation of illness to course and outcome, at every stage are influenced by cultural issues. Large numbers of patients get referred to the physician or psychiatrist of their cultural milieu as he/she can understand the patient and his psyche due to the understanding of cultural factors which influence the disease and healing process.

No culture confers absolute immunity against psychological vicissitudes. The forms of psychiatric disorders are identical in all cultures though the content of symptoms differ. For example, an Indian peasant when deluded complains of being possessed by a demon, while his western counterpart believes that his mind is being manipulated by electronics. It was believed a few decades ago, that people from oriental cultures experienced little or no stress. Mental illness and stress-related disorders like heart disease, high blood pressure, diabetes, cancer and suicide behavior were considered to be less frequent amongst them. This really is not so. Transcultural studies indicate that populations, exposed to a rapid onslaught from other cultures experience a cultural shock resulting in a high degree of mental and social stress.[1]

### Understanding of mental health in different cultures

Conceptually, if we look at the ancient culture, there are four cultural streams that are prominently seen. The Indian, Egyptian, Roman and Chinese culture. One similar phenomenon observed regarding mental health problems in all of them is the impact of supernatural on the human mind. The understanding of illness also in different cultures interestingly has been perceived as an imbalance of humors leading to problems of mind and body. For example, the personality traits sat, raj and tam and the three humors, vat, pitt and kaph conceptualized in Indian subcontinent also correspond to theories of Chinese and Roman culture. All cultures developed independently, thousands of miles apart with very little communication. The similar thinking about mental health issues shows the similarity of human thoughts across cultures.

In India, mental health and psyche has been an area of exploration for centuries together, right from the vedic period, there has been a description of human mind, its functioning, consciousness and dynamics of human behavior.

There have been a sizeable number of studies which relate to the demographic factors, cultural factors influencing presentation of illness, diagnosis of the illness-culture bound syndromes and influence of the cultural factors and the belief system on psychopathology, stigma and discrimination towards the patient. An attempt has been made to critically look at the research on culture and psychiatry in different areas and their influence on the patient, his diagnosis and treatment.

### Demographics

Trivedi JK *et al*.[3] in their study “Rapid urbanization - Its impact on mental health: A South Asian perspective” suggested that urbanization is affecting the entire gamut of population especially the vulnerable sections of society. Rapid urbanization has also led to creation of “fringe population” mostly living from hand to mouth which further adds to poverty. Urban population is heavily influenced by changing cultural dynamics leading to particular psychiatric problems like depression, alcoholism, and delinquency. Judicious use of resources, balanced approach to development, and sound government policies are advocated for appropriate growth of advancing economies of South-Asian region. Paralikar V *et al*.[4] while studying “Prevalence of clinically significant functional fatigue or weakness in specialty outpatient clinics of Pune, India.” found that overall prevalence of such disorders is 5.02% with higher rates in dermatology and ayurvedic clinics and a notable (63.83%) female preponderance.

### Presentation of symptoms in different cultures

Gautam and Kapur[5] in a study of psychiatric patients presenting with somatic complaints reported that more patients from Muslim ethnic group presented with somatic symptoms in South Indian population. Headache followed by nauseating sensation and vomiting were the prominent somatic complaints of the neurotic disorders. Gautam *et al*.[6] repeated the study in north Indian population and found that the predominant somatic complaint was constipation and feeling of gas in the abdomen.

Chaturvedi and Bhugra[7] reported that there has been a significant alteration in the concept of neurosis in most culture, with the relative abandonment of the term ‘neurosis’ and replacing the concept with that of common mental disorders, however, other conceptual equivalents of neurosis are seen in somatoform disorders, somatization and abnormal illness behaviour. Some traditional culture-bound neurotic syndromes and idioms of distress persist. Kulhara and Chakrabarti[8] studied “Culture and schizophrenia and other psychotic disorders” and observed that there is certain uniformity to the way schizophrenia presents globally; there are equally significant cultural differences. The outcome of schizophrenia appears to be better in developing, than developed cultures; reasons for this are far from clear, nevertheless, it can be safely assumed that culturally-determined processes, whether social or environmental, are partly responsible.

Jacob KS[9] reported in their study, “The cultures of depression.” that there are many cultural issues that need to be resolved. Clinically, there is a need to look beyond symptoms and explore personality, life events, situational difficulties and coping strategies in order to comprehensively evaluate the role of vulnerability, personality factors and stress in the causation of depression. In a review, “Depression among women in the South-Asian region: The underlying issues.”, Trivedi JK *et al*.[10] gave indications of specific health care needs of women in the region and suggested that mental health needs to be customized as per local needs and cultural sanctions.

### Culture-bound syndromes

Sumathipala A *et al*.[11] in their study “Culture-bound syndromes: The story of dhat syndrome.” explored the possibility of the presence of similar symptoms and syndromes in different cultures and historical settings. And concluded that the presence of similar symptoms and syndromes in different cultures and historical settings. Chaturvedi SK *et al*.[12] in their work, “ Dissociative disorders in a psychiatry institute in India - A selected review and patterns over a decade” emphasized that unlike in the West, dissociative identity disorders were rarely diagnosed; instead, possession states were commonly seen in the Indian population, indicating cross-cultural disparity.

Pereira S *et al*.[13] in their review “Making sense of ‘possession states’: Psychopathology and differential diagnosis.” presented clinical guidelines for a culturally sensitive assessment and management. Bhatia MS[14] in his study, “Compulsive spitting - A culture bound symptom “explored the possibility of compulsive spitting being a culture-bound symptom. Bhatia MS[15] also reported an analysis of 60 cases of culture bound syndromes.

Bhatia and Choudhary[16] described the contradictions in the diagnosis of hysteria in their paper “Hysteria - A chameleon or a fossil?”

### Cultural attitude to treatment

Saravanan B *et al*.[17] in their paper “Assessing insight in schizophrenia: East meets West.” concluded that the relationship between insight, awareness of illness and other clinical variables is similar in South India to elsewhere. However, the assessment of insight might have failed to capture locally accepted explanatory frameworks. Nunley M[18] in his study, “Why psychiatrists in India prescribe so many drugs?” offered the need to “sell” psychiatry as a legitimate kind of medicine by satisfying client expectations, and psychiatrists’ relationship to other actors in India’s pluralistic medical system, as factors that encourage a reliance on pharmaceutical or somatic interventions in psychiatric settings.

While describing her “Experiences with psychotherapy training in India.” Hoch[19] tried to show how difficulties encountered not only in psychotherapy with Indian patients, but also in supervision of candidates in training for psychotherapy can be related to specific cultural patterns of personality development and social intercourse and, beyond this, traced back to their deeper roots in the traditional Indian world view. In their paper, “Treatment of mental disorders in India”, Bagadia VN *et al*.[20] described the status and priorities of mental health in India. Rajkumar AP *et al*.[21] suggested that interventions after disaster should be grounded in ethno-cultural beliefs and practices and should be aimed at strengthening prevailing community coping strategies.

Phillips PA[22] did an exploratory study on dual diagnosis with staff perception of substance misuse among mentally ill of north India as the point of focus and reported that Dual diagnosis was seen as a common problem according to staff interviewed, although types of substance use reported were different than in western studies. Traditional substance use (the use of substances in distinct cultural, religious, and social settings that is not prohibited, such as khat or betel nut) also was reported as common among those with dual diagnosis.

Aggarwal NK[23] explored the identity, culture, and suffering with a Kashmiri Sikh refugee.

### Belief system influencing course and outcome

Shankar BR *et al*.[24] while studying “Explanatory models of common mental disorders among traditional healers and their patients in rural south India”, found that different terms, concepts and treatments were used by traditional and faith healers. 42.3% satisfied the International Classification of Diseases-10 Primary Care Version criteria for Common Mental Disorders. Mixed anxiety depression was the most common diagnosis (40%). they concluded that an understanding of local patient perspectives of common mental disorders will allow modern medicine to provide culturally sensitive and locally acceptable health care.

Saravanan B *et al*.[25] explored “Belief models in first episode schizophrenia in South India” and concluded that patients with schizophrenia in this region of India hold a variety of non-medical belief models, which influence patterns of health seeking and are likely to be rated as having less insight.

Bhugra D[26] described “Sati: A type of non-psychiatric suicide” and illustrated cultural factors, which may be seen as contributing to the act of suicide. Loganathan and Murthy SR[27] studied “Experiences of stigma and discrimination endured by people suffering from schizophrenia” and found significant differences between rural and urban respondents. They concluded that mental health programs and policies need to be sensitive to the consumer need and to organize services and to effectively decrease stigma and discrimination. Gautam S *et al*.[28] in a prospective study carried out to find relevance of stigma in North Indian population reported that with advancement in treatment, the themes of stigma are changing. The tools available to study the stigma have become obsolete and a culturally relevant tool, Jaipur Stigma Questionnaire designed, standardised and validated in the given population revealed that segregation of mentally ill from the society and shame for consultation are fading themes of stigma in the present time. Stigma is important in making access to health care difficult. The culturally relevant issues leading to stigma in north Indian population are social distance, rejection, guilt and responsibility for illness.

### Implications in psychotherapeutic process

In Indian thought, human behavior has been explored at length. In post-vedic period, in Upanishads, Bhagwad Gita, Yogic and ayurvedic literature abnormalities of human behavior have been described and the treatise has been emphasized mainly through psychic changes.[29] In India psychotherapy also needs to be based on cultural concepts and the prevailing belief system through centuries from generation to generation, which becomes more acceptable to the patient. If we accept psychotherapy as a interpersonal method of mitigating suffering, the process of change occurs in an individual through a psychotherapeutic relationship which has been described as the ‘guru- chela relationship’ in India, where in the wise offers advice to the pupil and helps him in relieving the suffering. This has been observed in Buddhist and Jain traditions too.

### Relevance to modern psychiatry

In the recent past there has been lot of research on use of many eastern techniques of healing in health sciences. Lot of emphasis is being laid on life style and health. Modern era and its increasing stresses call for stress management techniques and medicines devoid of side effects which increase the importance of alternative methods of medicine.

Scientific research on transcendental meditation programme has shown effectiveness of meditation on reducing neuroticism (Ander Tjoa) improving learning (Miskiman), improving academic achievements, prevention of alcohol (Shafii) and drug abuse (David Katz) There have been several reports on effect of transcendental meditation on reduction of anxiety, neuroticism (Jean Ross). Alexander and Schnieder reported comprehensive effects on neuroendocrine, psychological, social and spiritual factors related to substance abuse. Role of yoga in stress and sleep management, improving performance in sports and executives is being stressed recently. Prekshyadhyan a combination of meditation and relaxation technique has been found useful in improvement of concentration, memory and anxiety reduction in a study conducted at Jaipur by the author. Effectiveness of vipassana meditation as a therapeutic tool in psychological and psychosomatic illnesses has been reported by lyer and Flechman.

Some Ayurvedic combinations have been used as anti-anxiety and anti-depressants, reports of which are available from National Institute of Ayurveda, Jaipur. Vacha (*Acorus calamus*) and Jyotishmati (*Celastrus Panniculatus*) were found useful in treatment of depression (Bahetra). Unmad Bhanjan Ras a combination of 24 compounds was found to have anti-psychotic effect equivalent to chlorpromazine.

While communicating with cancer patients it was found by Gautam and Nijhawan,[30] that Indian patients tend to accept the diagnosis of cancer rather easily. The concept of death prevalent in Indian culture based on philosophy of Gita where soul is accepted as immortal and it is believed to transfer through death from one to another human/species plays a significant role in the easy acceptance of the diagnosis and the planning for the rest of the life.

The understanding of human psyche in vedantic model is more acceptable to Indian patients because of transfer of attitudes from generation to generation. Anecdotes from Bhagwat Gita as a psychotherapy of dying patient is virtually a tradition in Indian culture. Even now in many families when death is anticipated preaching of Lord Krishna stating that thoughts at the time of death determine the species of next birth help the individual to accept the death in a more gracious manner. The concept of “sthit pragna” 
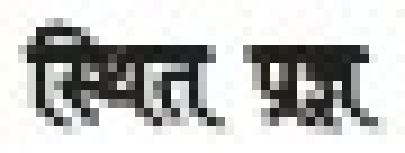
 how a person can remain detached from pleasure and sorrow unaffected by losses and gains inculates peace in the mind. One of the Neeti Shlokas says. 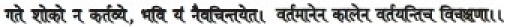


“It is not your duty to grieve the past nor should you worry about the future. Only he, who lives the present and thinks about the present is a wise man”, can help a depressed patient worrying for a recent loss.

Similarly many other anecdotes from ancient literature like Ramayana, Mahabharata and later writings like Neeti Shlokas and Panchatantra can be very usefully employed in supportive psychotherapy. There is a need to re-explore this vast treasure of knowledge which may be culturally relevant and useful for Indian patients. What is needed is to make our patients aware of their hidden potentials as was done by Jamwant to Hanuman before going to Lanka in the epic of Ramayan. These ancient texts should be re-explored for models of conflict resolution, understanding psychopathology and attainment of self-realization.

## CONCLUSION

Cultural factors influence understanding, presentation, diagnosis, management, course and outcome of mental illnesses. There is a need for culturally oriented modules of non-pharmacological management.

It would be appropriate to conclude with the words of Dr. Radhakrishnan: “India has seen empires come and go, has watched economic and political systems flourish and fade. It has seen these happen more than once. Recent events have ruffled but not diverted the march of India’s History. The culture of India has changed a great deal and yet has remained the same over three millennia. Fresh springs bubble up, fresh streams cut their own channels through the landscape, but sooner or later each rivulet, each stream merges into one of the great rivers which has been nourishing the Indian soil for centuries.”
